# Supplementary material for: Evidence of positive selection and a novel phylogeny among five subspecies of song sparrow (Melospiza melodia) in Alaska
Source: PeerJ. 2025 Oct 13;13:e19986. doi: 10.7717/peerj.19986 (PMC12530203; doi:10.7717/peerj.19986)
Supplement: Supplemental Information 2 — UAM = University of Alaska Museum. All sequences generated in this study are archived under SRA BioProject PRJNA1114297. † denotes individual used as a reference sequence. [file peerj-13-19986-s002.docx]

| Species | Locality | Catalogue Number | Tissue Number | Sequence Archive |
| --- | --- | --- | --- | --- |
| *Junco hyemalis*† | Virginia: Giles County, Mount Lake Biological Station | MLZ:Bird:69236 | n.a. | PRJNA493001 |
| *Melospiza georgiana* | Wisconsin: Burnett County, Saint Croix National Riverway | UAM11868 | CLP550 | SAMN41480621 |
| *Melospiza lincolnii* | Alaska: Southeast, Gravina Island | UAM21920 | ABJ048 | SAMN41480622 |
| *Melodia melodia maxima* | Alaska: Aleutian Islands, Attu Island | UAM31500 | UAMX4047 | PRJNA511035 |
| *Melospiza melodia maxima* | Alaska: Aleutian Islands, Adak Island | UAM10946 | CLP108 | SAMN41480625 |
| *Melospiza melodia sanaka* | Alaska: Shumagin Islands, Popof Island | UAM11585 | CLP251 | SAMN41480627 |
| *Melospiza melodia insignis* | Alaska: Kodiak Island, Cape Chiniak | UAM14002 | DDG1900 | SAMN41480624 |
| *Melospiza melodia caurina* | Alaska: Copper River Delta | UAM11384 | CLP020 | SAMN41480623 |
| *Melospiza melodia rufina* | Alaska: Southeast, Hyder | UAM7343 | KSW1374 | SAMN41480626 |
