# Supplementary material for: Evidence of positive selection and a novel phylogeny among five subspecies of song sparrow (Melospiza melodia) in Alaska
Source: PeerJ. 2025 Oct 13;13:e19986. doi: 10.7717/peerj.19986 (PMC12530203; doi:10.7717/peerj.19986)
Supplement: Supplemental Information 3 [file peerj-13-19986-s003.docx]

| Associated Phenotypic Trait | Gene | Accession Number |
| --- | --- | --- |
| Bill size | *ALX1* | XM_063155917.1 |
| Bill size | *CCDC149* | XM_063157733.1 |
| Bill size | *DLK1* | XM_063159381.1 |
| Bill size | *HMGA2* | XM_063155119.1 |
| Bill size | *LGI2* | XM_063157734.1 |
| Body size | *AFAP1* | XM_063157562.1 |
| Body size | *LAP3* | XM_063157687.1 |
| Body size | *LCORL* | XM_063157703.1 |
| Body size | *QDPR* | XM_063158102.1 |
| Body size | *SLIT2* | XM_063157711.1 |
| Body size | *WAPL* | XM_063163992.1 |
| Dispersal | *KCTD21* | XM_063148726.1 |
| Dispersal | *SLC2A1* | XM_063176803.1 |
| Dispersal | *TGFB2* | XM_063152222.1 |
| Migration | *CLOCK* | XM_063157256.1 |
| Migration | *CREB1* | XM_063162355.1 |
| Migration | *CRY1* | XM_063154387.1 |
| Migration | *CRY2* | XM_063159016.1 |
| Migration | *NPAS3* | XM_063160389.1 |
| Migration | *PER2* | XM_063166512.1 |
| Migration | *PER3* | XM_063177061.1 |
| Migration | *YPEL1* | XM_063173369.1 |
| Plumage color-carotenoid | *APOD1* | XM_063166976.1 |
| Plumage color-carotenoid | *BCO1* | XM_063168075.1 |
| Plumage color-carotenoid | *BCO2* | XM_063178621.1 |
| Plumage color-carotenoid | *CD36* | XM_063155614.1 |
| Plumage color-carotenoid | *HPS5* | XM_063158592.1 |
| Plumage color-carotenoid | *STARD3* | XM_063179094.1 |
| Plumage color- melanin | *AGRP* | XM_063167565.1 |
| Plumage color- melanin | *ASIP* | XM_063172145.1 |
| Plumage color- melanin | *MC1R* | XM_063168224.1 |
| Plumage color- melanin | *MITF* | XM_063165013.1 |
| Plumage color- melanin | *PCSK2* | XM_063152273.1 |
| Plumage color- melanin | *TYR* | XM_063150455.1 |
| Salt tolerance | *MMP17* | XM_063173281.1 |
| Salt tolerance | *MYOF* | XM_063163500.1 |
| Salt tolerance | *WNK2* | XM_063181827.1 |
