# Supplementary material for: Evidence of positive selection and a novel phylogeny among five subspecies of song sparrow (Melospiza melodia) in Alaska
Source: PeerJ. 2025 Oct 13;13:e19986. doi: 10.7717/peerj.19986 (PMC12530203; doi:10.7717/peerj.19986)
Supplement: Supplemental Information 4 — Subspecies sharing the same letter are not significantly different ( p > 0.05). [file peerj-13-19986-s004.docx]

|  | *M. m. maxima* | *M. m. sanaka* | *M. m. insignis* | *M. m. caurina* | *M. m. rufina* |
| --- | --- | --- | --- | --- | --- |
| Mass (g) | 46.2 ± 2.71 (A) | 45.4 ± 2.06 (A) | 41.0 ± 2.61 (B) | 28.4 ± 1.77 (C) | 27.9 ± 1.75 (C) |
| Wing chord length (mm) | 82.7 ± 3.04 (A) | 83.3 ± 2.45 (A) | 80.9 ± 2.14 (A) | 72.6 ± 1.43 (B) | 69.7 ± 3.03 (C) |
| Tail length (mm) | 78.9 ± 4.27 (A) | 77.8 ± 4.28 (A) | 75.4 ± 4.52 (A) | 67.1 ±4.28 (B) | 65.8 ± 3.01 (B) |
| Tarsus length (mm) | 27.6 ± 2.04 (A) | 27.0 ± 0.99 (A) | 26.3 ± 0.53 (AB) | 24.2 ±1.32 (BC) | 24.2 ± 1.41 (C) |
| Bill length (mm) | 12.4 ± 1.34 (A) | 12.4 ± 0.51 (A) | 11.9 ± 0.57 (AB) | 10.7 ± 0.60 (B) | 10.8 ± 2.75 (B) |
| Bill height (mm) | 7.5 ± 1.08 (A) | 6.89 ± 1.04 (B) | 6.5 ± 0.22 (AB) | 6.1 ± 0.45 (AB) | 6.37 ± 0.87 (B) |
| Bill width (mm) | 5.9 ± 0.77 (A) | 5.6 ± 0.56 (B) | 5.3 ± 0.32 (BC) | 5.1 ± 0.34 (BC) | 5.1 ± 0.45 (C) |
| Skull length (mm) | 38.8 ± 1.54 (A) | 38.8 ± 0.80 (A) | 38.0 ± 0.95 (A) | 35.7 ± 1.93 (B) | 34.8 ± 1.41 (B) |
