# Supplementary material for: Evidence of positive selection and a novel phylogeny among five subspecies of song sparrow (Melospiza melodia) in Alaska
Source: PeerJ. 2025 Oct 13;13:e19986. doi: 10.7717/peerj.19986 (PMC12530203; doi:10.7717/peerj.19986)
Supplement: Supplemental Information 5 [file peerj-13-19986-s005.docx]

|  | PC1 | PC2 | PC3 | PC4 | PC5 | PC6 | PC7 | PC8 |
| --- | --- | --- | --- | --- | --- | --- | --- | --- |
| MASS | -0.44967 | -0.00369 | -0.02 | 0.088395 | 0.044583 | -0.0053 | 0.593971 | 0.659361 |
| WCH | -0.44058 | 0.04157 | 0.079172 | 0.134248 | 0.2834 | -0.13608 | 0.41728 | -0.71198 |
| TL | -0.41001 | 0.008032 | 0.111733 | 0.102829 | 0.19855 | -0.66887 | -0.53913 | 0.176898 |
| TS | -0.34709 | 0.032705 | 0.364038 | 0.01502 | -0.85266 | 0.036712 | -0.05591 | -0.11918 |
| BL | -0.22811 | 0.641445 | -0.38509 | -0.61816 | -0.04983 | 0.006027 | -0.05086 | -0.03156 |
| BLH | -0.24041 | -0.67853 | 0.079709 | -0.66597 | 0.107266 | 0.125813 | -0.0628 | -0.02572 |
| BLW | -0.23356 | -0.31855 | -0.81763 | 0.300145 | -0.23697 | 0.060322 | -0.13138 | -0.09125 |
| SKL | -0.38824 | 0.154282 | 0.158645 | 0.21828 | 0.284774 | 0.716389 | -0.39433 | 0.05337 |
