# Supplementary material for: Evidence of positive selection and a novel phylogeny among five subspecies of song sparrow (Melospiza melodia) in Alaska
Source: PeerJ. 2025 Oct 13;13:e19986. doi: 10.7717/peerj.19986 (PMC12530203; doi:10.7717/peerj.19986)
Supplement: Supplemental Information 6 [file peerj-13-19986-s006.docx]

| Taxa | Tissue Number | Number of reads | % properly paired reads | Mean depth of coverage |
| --- | --- | --- | --- | --- |
| *Melospiza georgiana* | CLP550 | 81,339,509 | 93.0% | 7.04 |
| *Melospiza lincolnii* | ABJ048 | 12,566,699 | 92.9% | 2.10 |
| *Melospiza melodia maxima* | CLP108 | 26,989,233 | 91.4% | 3.33 |
| *Melospiza melodia sanaka* | CLP251 | 84,504,717 | 94.0% | 9.71 |
| *Melospiza melodia insignis* | DDG1900 | 73,078,606 | 92.0% | 8.62 |
| *Melospiza melodia caurina* | CLP020 | 69,765,460 | 92.0% | 8.12 |
| *Melospiza melodia rufina* | KSW1374 | 72,593,062 | 90.6% | 8.08 |
