# Supplementary figures and images for: Evidence of positive selection and a novel phylogeny among five subspecies of song sparrow (Melospiza melodia) in Alaska
Source: PeerJ. 2025 Oct 13;13:e19986. doi: 10.7717/peerj.19986 (PMC12530203; doi:10.7717/peerj.19986)

**RUSSIA**

**CANADA**

**ALASKA**

← 2.19 | 1.75 →

← 1.37 | 5.07 →

← 2.90 | 1.20 →

← 3.96 | 2.37 →

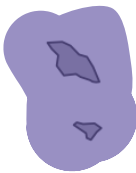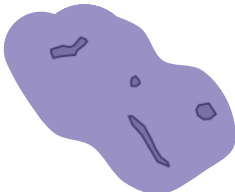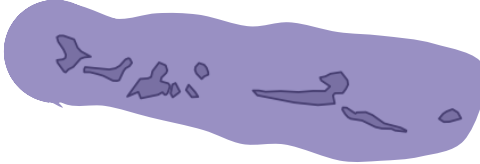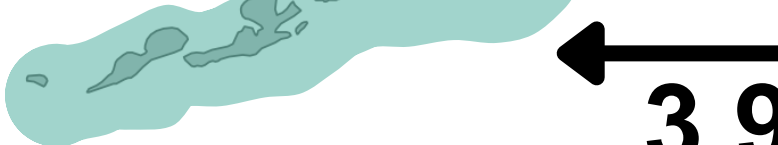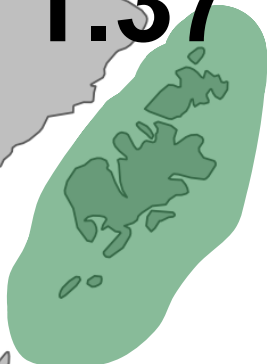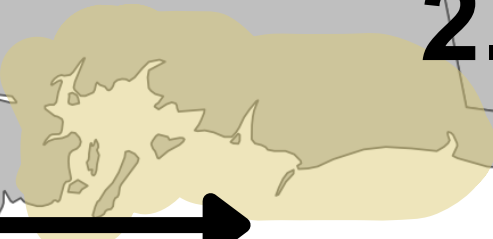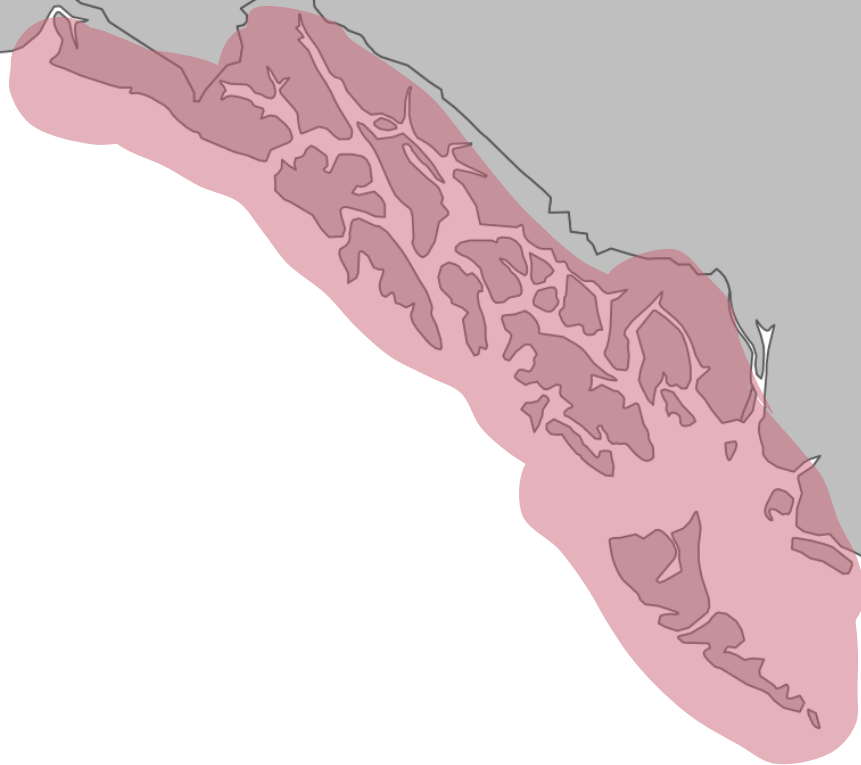

Supplement: Supplemental Information 7 — Values denote the number of migrant individuals per generation relative to effective population size, and the arrows denote direction of migration. For example, M. m. maxima is receiving 2.90 migrants per generation from M. m. sanaka. Colors correspond to Figure 1. [file peerj-13-19986-s007.pdf]
